# Effects of moxibustion for obesity: A protocol for systematic review and meta-analysis

**DOI:** 10.1097/MD.0000000000027910

**Published:** 2021-12-03

**Authors:** Hyungsuk Kim, Koh-Woon Kim, Won-Seok Chung

**Affiliations:** a Department of Korean Medicine Rehabilitation, Kyung Hee University Medical Center, 23 Kyungheedae-ro, Dongdaemun-gu, Seoul, Korea; b Department of Korean Rehabilitation Medicine, College of Korean Medicine, Kyung Hee University, Seoul, Korea.

**Keywords:** meta-analysis, moxibustion, obesity, protocol, systematic review

## Abstract

**Background:**

Obesity is a chronic disease associated with lethal health conditions. Moxibustion, a frequently used treatment in traditional Chinese medicine, is effective and safe for the treatment of obesity. However, the evidence has not been systematically collected and combined to date. This systematic review and meta-analysis will analyze the effects of moxibustion on obesity.

**Methods:**

The following databases will be searched: Cochrane Central Register of Controlled Trials, MEDLINE/PubMed, EMBASE, 1 Chinese database (Chinese National Knowledge Infrastructure), 1 Japanese database (Citation Information by the National Institute of Informatics), and 3 Korean databases (Oriental Medicine Advanced Searching Integrated System, ScienceON, and KoreaMed). The quality of the included studies will be assessed according to the Cochrane Assessment Tool for Risk of Bias. Data from the included studies will be synthesized for meta-analysis. The primary outcome will be body weight, and the secondary outcomes will be body mass index, waist-hip ratio, waist circumference, hip circumference, and effective rate.

**Results and Conclusion:**

Ethical approval is not necessary for this study because it will not include any patient information. The results of this systematic review and meta-analysis will be publicly available and published in a peer-reviewed journal.

**Registration number:**

DOI 10.17605/OSF.IO/NTKDF (https://osf.io/ntkdf).

## 1 Background

Obesity is considered a chronic disease globally. The prevalence of obesity is increasing in many developed countries.^[1–3]^ It is associated with several other diseases affecting the various systems in the body, such as the cardiovascular,^[4]^ pulmonary,^[5]^ and musculoskeletal systems. Furthermore, obesity is associated with a high mortality rate from any cause.^[6]^ Therefore, successful management of obesity contributes to overall health.

Many studies have aimed to suggest interventions to treat obesity.^[7]^ Lifestyle intervention is the primary consideration for the treatment of obesity. The main components include a limited diet, proper exercise, and behavioral modifications.^[8]^ A good attendance and adherence of the patients to the treatment regimen is key to success,^[9]^ and contact with medical professionals to maintain motivation may be required. Moreover, drug therapies are known to be efficacious and are considered for patients with a body mass index (BMI) of 27 kg/m^2^ and weight-related diseases or a BMI of 30 kg/m^2^.^[10]^ Some medications are effective for the treatment of obesity; however, their adverse events should be closely observed by doctors to ensure safety.^[11]^

Moxibustion, a traditional Chinese medicine (TCM) approach, is a type of heat therapy that uses herbs such as mugwort, which is burned by fire to transmit heat sensation to a patient's skin.^[12]^ The adverse events include burns during the procedure due to failure of controlling the heat; however, it is considered a relatively safe intervention.^[13]^ Moxibustion has been used in various medical conditions, such as lumbar disc herniation,^[14]^ ankylosing spondylitis,^[15]^ insomnia,^[16]^ and constipation. Moreover, moxibustion is affects metabolic diseases such as diabetes^[17]^ and many clinicians use moxibustion in clinical situations for the treatment of obesity. However, systematic reviews and meta-analyses have not been conducted thus far.

In this protocol, we have suggested a scheme for the systematic review and meta-analysis of moxibustion for the treatment of obesity to evaluate its efficacy.

## 2 Methods

### 2.1 Study registration

This protocol is written in accordance with the Preferred Reporting Items for Systematic Reviews and Meta-Analysis protocols.^[18]^ The protocol of this systematic review is uploaded and registered at the Open Science Framework (osf.io/ntkdf).

### 2.2 Eligibility criteria for study selection

#### 2.2.1 Types of studies

Randomized controlled trials (RCTs) will be selected and analyzed in this systematic review. Quasi-RCTs or crossover studies will not be considered for review in this study. RCTs published in any language will be accepted as long as it is retrieved from the databases.

#### 2.2.2 Types of participants

Patients diagnosed with obesity will be included in this study. Patients of any age and sex will be considered without any restrictions.

#### 2.2.3 Types of interventions

The experimental group will be treated with moxibustion therapy. Materials to protect the skin from coming in direct contact with heat may be used. If a needle is used with heat, that is, warm needle therapy, it will not be accepted as an intervention in this review. Moxibustion combined with other therapies will be acceptable as long as the same composition of interventions are included in the control group, except for moxibustion itself. Combined therapy can include lifestyle modifications, conventional drugs, and other TCM interventions. The control group could also be a placebo control group.

#### 2.2.4 Types of outcome measures

The primary outcome will be body weight. The secondary outcomes will be BMI, waist-hip ratio, waist circumference, hip circumference, and effective rate.

### 2.3 Search strategy

#### 2.3.1 Electronic data

The Cochrane Central Register of Controlled Trials, MEDLINE/PubMed, EMBASE, 1 Chinese database (Chinese National Knowledge Infrastructure), 1 Japanese database (Citation Information by the National Institute of Informatics), and 3 Korean databases (Oriental Medicine Advanced Searching Integrated System, ScienceON, and KoreaMed) will be systematically searched from their inception to March 2021. The search methods for PubMed are presented in Table 1.

**Table 1:** Search strategy for PubMed.

“obes∗” [All Fields] OR “weight gain∗”[All Fields] OR “weight loss”[All Fields] OR “body mass ind∗”[All Fields] OR “adipos∗”[All Fields] OR “overweight”[MeSH Terms] OR “overweight”[All Fields] OR “overweighted”[All Fields] OR “overweightness”[All Fields] OR “overweights”[All Fields]) OR “over weight”[All Fields] OR “overload syndrome∗”[All Fields] OR “overeat∗”[All Fields] OR “over eat∗”[All Fields] OR “overfeed∗”[All Fields] OR “over feed∗”[All Fields] OR “weight cycling”[All Fields] OR “weight reduc∗”[All Fields] OR “weight los∗”[All Fields] OR “weight maint∗”[All Fields] OR “weight decreas∗”[All Fields] OR “weight watch∗”[All Fields] OR “weight control∗”[All Fields] OR “weight gain∗”[All Fields] OR “weight chang∗”[All Fields]) AND (moxibustion[All Fields] OR artemisia[All Fields] OR moxa∗[All Fields]) AND (“randomized controlled trial”[All Fields] OR “controlled clinical trial”[All Fields] OR “random∗”[All Fields] OR “placebo”[All Fields] OR “trial”[All Fields]

#### 2.3.2 Search for other resources

To search for related articles more completely, we will scan the reference lists of the included articles. For offline-only materials, manual searching by researchers will also be conducted.

### 2.4 Data collection and analysis

#### 2.4.1 Study selection

**Figure 1:**
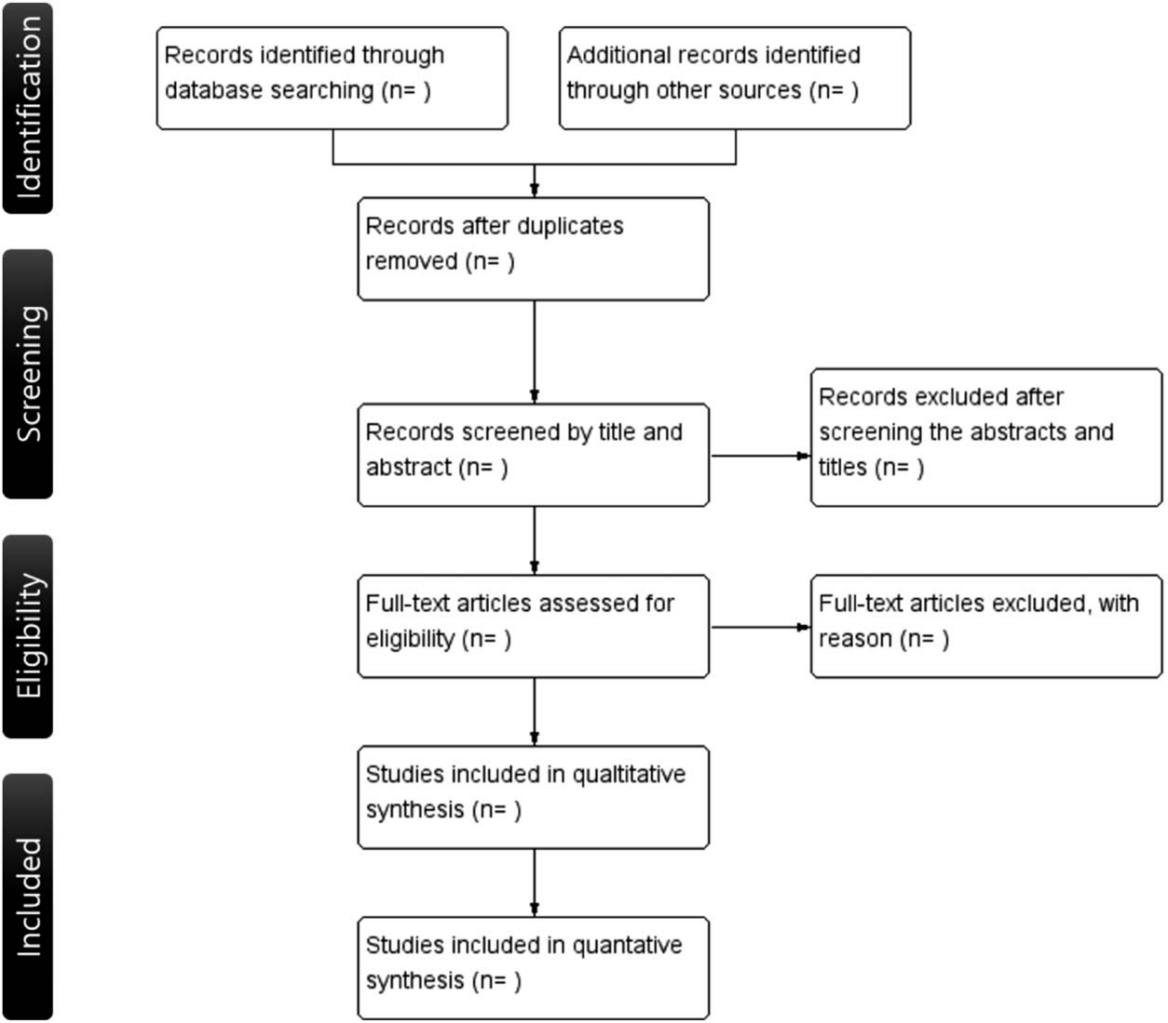
Flow diagram of the systematic review.

Two independent researchers will search databases and other sources individually according to a search guideline. The specific contents of the guidelines for the inclusion and exclusion criteria with examples will be provided to them for article selection. When there will be inconsistency between the reviewers, a third researcher will make a decision by evaluating both opinions. A flow diagram of the selection process is shown in Figure 1.

#### 2.4.2 Data extraction and management

A previously manufactured Excel sheet will be used to help reviewers extract data from included studies in a discreet manner. The items on the sheet will be the name of authors, intervention group and comparison group with the number of patients, details of interventions, frequency and duration of treatment, outcome measurement with the kind and time point of measurement, results of outcomes, and adverse events. If the data are not clear or 2 datasheets show different contents, another reviewer will make a decision.

#### 2.4.3 Assessment of the risk of bias and quality of the included studies

Two reviewers will assess the risk of bias in 7 domains independently using the tool made by the Cochrane Collaboration group.^[19]^ The domains are as follows: random sequence generation, allocation concealment, blinding of participants and personnel, blinding of outcome assessment, incomplete outcome data, selective outcome reporting, and other sources of bias. Each domain of risk of bias will be assessed by selecting 1 of 3 categories—high risk, low risk, or unclear risk. If the 2 reviewers have different opinions regarding the decision on the categories, another reviewer's opinion will be considered.

#### 2.4.4 Assessment of the effect of treatment

Continuous data on outcomes will be depicted as mean differences with 95% confidence intervals.

#### 2.4.5 Management of missing data

If data that we need are not included in the paper, we will send an e-mail to the corresponding author for more information. If the author refuses or does not answer our request, the missing data will not be considered for analysis in our study.

#### 2.4.6 Data synthesis

If data can be synthesized by the researchers, meta-analyses will be conducted using the software distributed by the Cochrane Collaboration (Review Manager Software Version 5.3, Copenhagen). In the case of *I*^2^ > 50%, the heterogeneity will be considered high, and a random-effects model will be used, whereas in the case of *I*^2^ < 50%, a fixed-effects model will be used. We will perform subgroup analysis when the heterogeneity is high and the division of the whole group according to a certain category is thought to be reasonable.

#### 2.4.7 Subgroup analysis

The criteria for subgroup analysis will be as follows:

- The type of interventions combined with moxibustion in the experimental group
- Duration of interventions
- Time-point of follow-up

#### 2.4.8 Ethics and dissemination

This is only a protocol for the study of systematic review and meta-analysis. Therefore, no patient information will be included in this article. Therefore, ethical approval was not required. The findings of this systematic review and meta-analysis will be written as a manuscript and submitted to a journal for dissemination of information.

## 3 Results and Conclusion

Obesity is one of the main interests of patients, researchers, and clinicians. This disease lowers the quality of life and threatens the overall health. Moxibustion, as a facet of TCM treatment, is known to be both safe and effective for the treatment of various diseases, and the increasing number of RCTs on obesity treatment with moxibustion have been published recently. The results of this systematic review and meta-analysis will synthesize the pre-existing evidence to benefit all those who treat and suffer from obesity.

## Author contributions

**Conceptualization:** Won-Seok Chung, Koh-Woon Kim.

**Data curation:** Hyungsuk Kim, Koh-Woon Kim.

**Formal analysis:** Hyungsuk Kim, Koh-Woon Kim, Won-Seok Chung.

**Funding acquisition:** Won-Seok Chung.

**Project administration:** Hyungsuk Kim, Koh-Woon Kim, Won-Seok Chung.

**Writing – original draft:** Hyungsuk Kim.

**Writing – review & editing:** Hyungsuk Kim, Won-Seok Chung.
